# Transdermal Drug Delivery of Tazarotene: Determining Tazarotene’s Potential in Local Transdermal Therapy

**DOI:** 10.3390/pharmaceutics16010064

**Published:** 2023-12-31

**Authors:** Helena Hamzehpour, Ástrós Óskarsdóttir, Helgi Jónsson, Fjóla Jónsdóttir, Ólafur E. Sigurjónsson, Bergthora S. Snorradottir

**Affiliations:** 1 Faculty of Pharmaceutical Sciences, University of Iceland, 107 Reykjavik, Iceland; heh83@hi.is (H.H.);; 2 Faculty of Medicine, University of Iceland, 107 Reykjavik, Iceland; helgi@hi.is; 3 Faculty of Industrial Engineering, Mechanical Engineering and Computer Science, University of Iceland, 107 Reykjavik, Iceland; fj@hi.is; 4 School of Science and Engineering, Reykjavik University, 102 Reykjavik, Iceland

**Keywords:** transdermal delivery, tazarotene, tape-stripping, diffusion, porcine, hand osteoarthritis

## Abstract

Retinoid-based drugs, while effective, are associated with systemic toxicity. Topical alternatives offer a safer option, and tazarotene, a third-generation synthetic retinoid, holds promise. This study investigates tazarotene’s transdermal delivery potential, focusing on its application for joint-related conditions. The aim of this study was to investigate the suitability of tazarotene as a candidate for transdermal delivery into joints. In vitro permeation studies, using porcine skin, assessed tazarotene’s transdermal drug delivery from solution and gel formulations. A tape-stripping analysis determined stratum corneum retention and a pilot study using porcine joints assessed tazarotene’s ability to reach articular cartilage. Ultra Performance Liquid Chromatography coupled with a mass detector method was used to quantify tazarotene and tazarotenic acid permeation. The results validate that tazarotene can permeate porcine skin and accumulate in articular cartilage in detectable amounts. The detection of tazarotene and tazarotenic acid in both the in vitro permeation studies and the pilot study on porcine joints validate the drug’s potential therapeutic use for hand osteoarthritis. This study lays the groundwork for future research, contributing insights into tazarotene’s potential for transdermal drug delivery and guiding further exploration in topical retinoid applications.

## 1. Introduction

Recent discoveries associating the retinoic acid pathway to osteoarthritis, more specifically hand osteoarthritis (HOA), have sparked a growing interest in exploring the potential of retinoids in addressing the unmet need for therapeutic interventions for the disease [1]. Retinoic acid (RA)-derived drugs, although widely used, are associated with considerable toxicity and side effects. These include mucocutaneous side effects, liver toxicity, teratogenic properties, and dyslipidemia [2,3]. The chronic use of systemic retinoids can also lead to bone abnormalities, resembling diffuse idiopathic skeletal hyperostosis. Unlike other side effects of retinoids, which can be reversible upon discontinuation, skeletal abnormalities may persist even after stopping the medication [4].

In contrast, topical retinoids offer a significantly safer alternative. Transdermal delivery with lower side effects and a better safety profile is favorable over systematic delivery. Local side effects, such as erythema, dryness, itching, and stinging, are more common during the early stages of treatment, manageable, and tend to subside with time [5]. For this reason, this study investigates if retinoids can be delivered transdermally to the articular cartilage of joints for local therapeutic intervention. Given that the penetration of RA through the skin is generally low [6], using a selective third-generation retinoid, such as tazarotene, could potentially increase the transdermal bioavailability of retinoic acid receptor agonists (RAR-agonist) in joints. 

Tazarotene, a third-generation synthetic retinoid, is known for its application in topical formulations for psoriasis, acne vulgaris, and photoaging [7,8]. Tazarotene is an acetylenic retinoid with a rigid structure due to a triple bond joining the aromatic rings. This rigid structure gives the drug its selective affinity for RARs, specifically RAR-β and RAR-γ, minimizing off-target binding and thus undesirable effects [9,10]. The drug has a low molecular weight of 351.5 g/mol making it suitable for transdermal delivery. Tazarotene’s logP value, on the other hand, is 5.96, making the drug highly lipophilic [11,12,13]. Following its topical application, tazarotene undergoes de-esterification within the body to its active metabolite, tazarotenic acid. This active metabolite is chiefly responsible for the therapeutic benefits associated with the drug [14]. When compared to adapalene, also a third-generation retinoid, tazarotene demonstrates greater potency, requiring less of the drug to attain the same desired outcome. This implies that tazarotene treatments can be less frequent or employ lower doses while still achieving equivalent therapeutic outcomes [15].

One of the main limitations of the transdermal drug delivery route is the permeability of the drug through the skin. In percutaneous drug absorption, the stratum corneum (SC) is believed to be the major rate-limiting step [16]. Permeation studies, often employing Franz diffusion cells, provide insights into drug absorption through the skin. Although human skin is ethically restricted, inbred animals like porcine, owing to their structural resemblance to human skin, are widely used [17]. Porcine ear skin has been identified as the most promising for Franz-diffusion cell studies, due to its similarities to human skin and its greater accessibility as compared to human skin, as well as being recommended by the OECD guidelines. The principle of the three R’s (replacing, reducing, and refining) aims to minimize the use of animals as best as possible [18], highlighting the ethical issue of reducing animal testing. Therefore, it is valuable that a by-product from a local abattoir can be used to determine transdermal drug delivery. 

With an emphasis on potential therapeutic interventions, the study first examines transdermal delivery through the outermost barrier of the stratum corneum and, second, conducts a pilot study on tazarotene drug delivery to joints in quantifiable amounts. The transdermal route has gained prominence due to its unique advantages, including the absence of a first-pass effect, gastrointestinal protection, prolonged drug delivery, and improved patient compliance [19]. Additionally, local treatment has the advantage of targeting the affected area, thus lowering drug dosages compared to systemic treatments and the risk of side effects. Local treatment of HOA, for instance, would have the benefit of targeting treatment towards symptomatic joints, as the disease activity is often restricted to a few joints at a given time [20,21]. In targeting the most affected joints and reducing the inflammatory state, the structural damage to the joint could be minimized. 

In this study, we focus on exploring the potential of tazarotene for transdermal delivery into joints. The main focus is on examining the diffusion of the drug through the skin and the quantification of the drug in articular cartilage. 

## 2. Materials and Methods

### 2.1. Materials

Tazarotene, tazarotenic acid, L-ascorbic acid, and sodium phosphate dibasic were purchased from Sigma Aldrich. Acetonitrile (HPLC grade), methanol (HPLC grade), formic acid (HPLC grade), and potassium phosphate monobasic were purchased from Riedel-de Haen. Ammonium acetate, sodium chloride, Carbopol, Ceta-stearyl alcohol, polyethylene glycol 6’000, propylene glycol, paraffin oil, and cyclodextrin were purchased from Merck, Emsure, BF Goodrich, Unikem, Fluka Analytical, Mecobenzon, Bufa B.V., and Wacker, respectively.

### 2.2. Preparation of Topical Formulations

For the transdermal drug release study, a tazarotene stock solution with a concentration of 5 mg/mL was prepared in isopropyl alcohol. The solution was diluted with receptor fluid, phosphate buffer saline (PBS, pH 7.4), and 2.5% cyclodextrin, for the study. Tazarotene gel was prepared according to Table 1, and the previously described stock solution was added to the formulation, at a concentration of 0.1%, just before the experiments started. 

For the pilot study, a gel and a cream base were prepared using the ingredients listed in Table 1. For preservation, 0.1% paraoxybenzoate (using a 10% diluted 4:1 methylparaben:propylparaben solution) was added to both bases. The tazarotene stock solution was added to the formulations, at a concentration of 0.1%, just before the experiments started. The pH range was 5–7.

### 2.3. Release Studies by In Vitro Experiments

The in vitro experiment was performed using porcine skin. Porcine skin is understood to have the highest structural resemblance to human skin compared to other inbred animal models and is considered more ethically correct to use [17,22,23]. Porcine ears were provided by Stjörnugrís (a local abattoir) from freshly killed 6-month-old animals, as the ears are often considered a by-product of their production. Before use, the ears were washed with distilled water and carefully dried. The full-thickness porcine skin was then carefully separated from the underlying cartilage and stored in aluminum foil at −20 °C (less than 3 months) until use. Before use, the porcine ear skin was thawed at room temperature, punched into 1.5 cm disks, and dermatomed to nominal thickness (ca. ≥1 mm) with a sterile scalpel blade [24]. The thickness of each disk was then determined using a caliper, followed by a visual analysis of the skin using optical microscopy as a means to eliminate damaged skin disks.

The in vitro permeation assays were carried out using unjacketed Franz diffusion cells with an inner diameter of 9.0 mm, an effective diffusion area of 0.64 cm^2^, a donor chamber volume of 1 mL, and a receptor chamber volume of 12 mL. The receptor chamber was filled with previously degassed receptor fluid, PBS pH 7.4 (prepared according to the European Pharmacopoeia guidelines), and 2.5% cyclodextrin, as previous studies [25,26] have shown that the addition can aid the solubility in the receptor phase. Dermatomed skin discs were immersed in PBS and fixed between the donor and receptor chambers with the epidermal side facing up towards the donor compartment. The diffusion cells were then sealed using a clamp and stationed on a magnetic stirrer plate inside an oven at a temperature of 32 ± 1 °C. The stirring speed was set to 400 rpm and the Franz diffusion cells were left to equilibrate for 30 min. 

The experiment was started when the test substances were placed into the donor compartments (1.0 mL of a tazarotene solution or 500 mg of the 0.1% tazarotene gel). Both the donor compartments and sampling port were sealed using paraffin film to prevent evaporation. Using a disposable syringe, 150 µL samples were collected from the sample port at predetermined time intervals: 11, 13, 15, 17, and 19 h for the solution and 2, 4, 6, 8, and 24 h for the gel. The removed volume was replaced with the equivalent volume of fresh receptor fluid. Data were collected from a minimum of four replicates for each experiment and samples were analyzed by UPLC-QDa to determine the quantity of permeated tazarotene and tazarotenic acid.

### 2.4. Tape-Stripping

After the completion of the release study, skin samples were removed from the Franz cells, rinsed with 2 mL of deionized water, and carefully dried. Adhesive tape strips were prepared in advance (thickness of 0.5–1 µm) and applied individually to the skin samples to remove the SC via the tape-stripping method. Uniform pressure was applied twice by gently rolling a 2 kg weight over the tape, after which the adhesive strip was removed using a sharp upward movement. The tape strips were then placed into 1.5 mL microtubes (2 strips per tube), and 1 mL of methanol was added to each tube. The pressure applied to the tape and the speed of removal should be consistent throughout the entire process to ensure uniformity in all samples. With each tape strip removed, a fraction of the SC is also removed. The SC is consecutively removed from the same area of the skin by repeated tape stripping. In total, each skin sample was stripped 70 times, equivalent to a skin thickness of 35–70 µm. The microtubes were then stored at 4 °C overnight (12 h), followed by 15 min sonication and 10 min centrifugation at 12,000 rpm. Aliquots of the extraction were quantified using UPLC-QDa analysis.

### 2.5. UPLC-QDa Analysis

Chromatographic separations were performed using a Waters Acquity UPLC system coupled to a Waters Acquity QDa mass detector. The separation was carried out on an Acquity UPLC^®^ BEH C18 (1.7 µm) column at 38 °C. Mobile phases A and B consisted of acetonitrile and 1% formic acid with 5mM ammonium acetate, respectively. At a flow rate of 0.4 mL/min, the gradient program was as follows: 0–2.1 min (85% A), 2.1–2.5 min (100% A), followed by re-equilibration from 2.5–3 min (85% A). The autosampler temperature was set to 10 °C while the injection volume was 5 µL. The QDa mass detector was operated in positive electrospray ionization (ESI) mode via selected ion recording (SIR) at 15V cone voltage to detect the analytes’ chromatographic peaks (Table 2). Data analysis and interpretation were performed using TargetLynx (version 4.2). 

### 2.6. Pilot Study Using Porcine Joints

**Figure 1:**
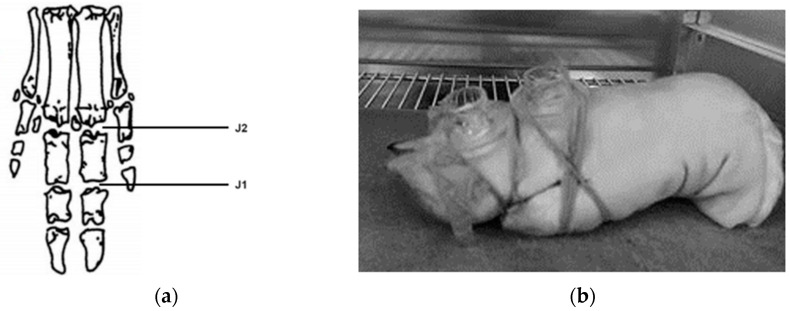
Porcine joint study setup. (**a**) The anatomical structure of porcine toes with joints J1 and J2 labeled. (**b**) The setup of the donor compartments for the study.

Another by-product of Stjörnugrís‘s abattoir operations is porcine toes, which were used to study the permeation of tazarotene into joints. Prior to use, the toes were washed with distilled water and dried. A gel and a cream mixture (500 mg) containing 0.1% tazarotene were placed in the donor compartments of Franz diffusion cells that had been securely positioned on two joints, J1 and J2, as shown in Figure 1. The compartments were then closed with parafilm to prevent evaporation. The porcine toes were placed in a fume hood at room temperature for 21–24 h. 

After 21–24 h, the articular cartilage was carefully collected and placed in microtubes. For extraction, 0.7 mL of methanol was added to each tube and the tubes were placed on a shaker for 20 h. The samples were then centrifuged for 10 min at 17,709× *g*. After centrifugation, 0.4 mL samples were taken from each tube, filtered, and analyzed via UPLC-QDa.

## 3. Results

### 3.1. Transdermal Drug Delivery

**Figure 2:**
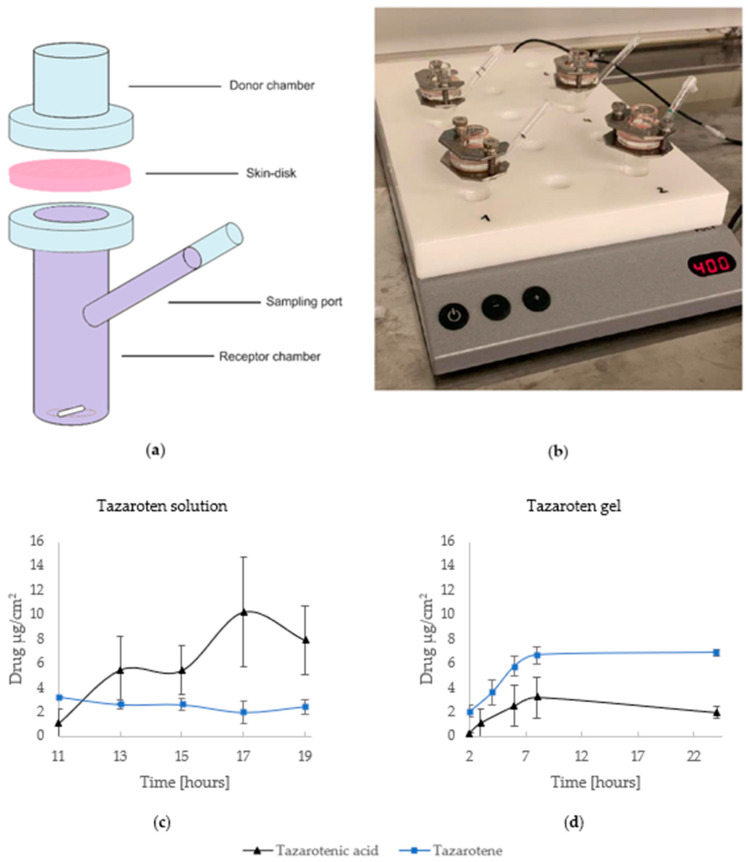
In vitro permeation study. (**a**) Illustration of unjacketed Franz diffusion cell. (**b**) Experimental setup of Franz diffusion cells on a magnetic stirrer plate. Below, the amount of drug release (μg/cm^2^) for tazarotene (**blue**) and tazarotenic acid (**black**) is displayed. (**c**) The average cumulative amount of drug released per hour using a tazarotene solution (n = 4). (**d**) The average cumulative amount of drug released per hour using a tazarotene gel (n = 4).

Experiments were carried out using a tazarotene solution (*n* = 4) as well as a tazarotene gel formulation (*n* = 4). The data, seen in Figure 2, show the drug release over time and validate that tazarotene can permeate the skin in detectable amounts both from a solution and a gel formulation. The detection of tazarotenic acid across the sampled specimens indicates the metabolic conversion of tazarotene during transdermal delivery. An additional peak was detected in some samples, with a retention time of 1.98 min and molecular weight of 279.2 g/mol, suggesting further metabolism of tazarotene and tazarotenic acid. 

When examining the concentrations of tazarotenic acid in both the solution and gel, distinct variations in activation kinetics were noted. The solution exhibited a more pronounced metabolic trend, as evidenced by the higher tazarotenic acid levels relative to tazarotene in the experiments using the solution, as opposed to the gel formulation.

### 3.2. Tape Stripping

**Figure 3:**
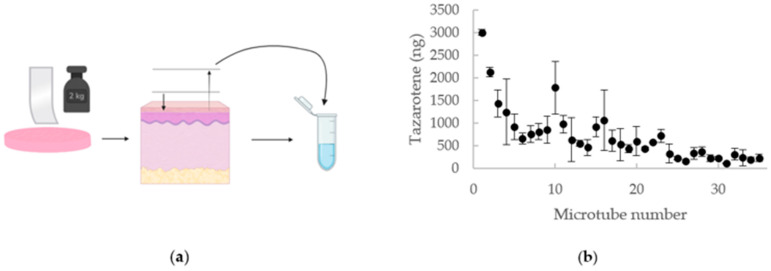
Tape Stripping. (**a**) Illustration of the tape-stripping method. (**b**) Tape-stripping data shown as the mean mass (•) of replicates ± standard deviation. Eppendorf-tube no.1 represents tape no. 1–2, etc.

The tazarotene concentration in the SC was determined using the tape-stripping method. The tape-stripping method can provide information about drug distribution within the SC. As presented in Figure 3, the amount of tazarotene in the SC decreased with increased depth into the skin. The total drug amount detected in all strips yields an approximate total of 0.025 mg, or 5% of the drug applied to the skin, retained within the SC. 

### 3.3. Pilot Study

Penetration experiments were performed on porcine joints, the first using 0.1% tazarotene gel (n = 2) and the second using 0.1% tazarotene cream (n = 2). Samples were collected from two joints for each replicate, the first and second joints of the porcine toe (Figure 1). The data collected show that the drug reached the articular cartilage, although only a fraction of the applied amount was detected. The mean mass of tazarotene and tazarotenic acid in each joint as well as the percentage of tazarotene that reached the joint can be seen in Table 3. The tazarotene concentration exhibited notably greater variability, but a slightly lower concentration of tazarotenenic acid with the gel formulation, implying that the cream formulation may be more suitable for the transdermal delivery of tazarotene.

## 4. Discussion

The release studies from the Franz diffusion cell experiment showed that tazarotene can permeate the skin in quantifiable amounts. The diffusion profile suggests that the oxidative metabolism of the drug occurred with its penetration through the skin. As previously mentioned, tazarotene is metabolized into tazarotenic acid, the active metabolite, which is further metabolized into sulfoxides, sulfones, and other polar metabolites [14]. These polar metabolites are not active and, thus, do not exhibit the same properties as tazarotenic acid [10]. Of the identified degradation products for tazarotene, one molecule, with its ester group removed, has a molecular weight of 280.1 g/mol. Fragmentation ions of other tazarotene degradation products can have the same molecular weight [27,28]. Consequently, the additional peak observed at 1.98 min likely represents one of the degradation products of tazarotene. As mentioned before, tazarotene was metabolized more in the solution in comparison to the gel formulation, as tazarotenic acid was detected in larger amounts when using the solution compared to the gel. A probable explanation for this is the addition of ascorbic acid in the base formulation of the gel, as ascorbic acid has antioxidant properties and can therefore reduce oxidative stress [29]. The in vitro permeation study for the tazarotene solution did not follow a typical profile, as permeation did not increase gradually with time. Various factors can affect the results, such as air bubbles forming beneath the skin and variability of the skin, as it can be from different animals [30]. Differences in skin characteristics within the species, such as skin thickness, hydration levels, and skin conditions, can lead to variations in drug permeation rates. Some variations may even influence the metabolism of the drug within the skin, due to different levels of enzymatic activities [31]. A small fraction of the drug penetrated the skin, and because tazarotene is a highly lipophilic compound (log*p* value > 5), it can be assumed that it is absorbed poorly through the skin. However, several approaches can be applied to enhance the bioavailability of topically applied drugs. The use of various permeation enhancers, for example, could increase the transdermal delivery of tazarotene [32]. Cyclodextrin (CD), for instance, could potentially be implemented in the base formulations as it increases the permeability of lipophilic drugs through the skin, improves their stability, and can reduce topical irritation of retinoids [33,34]. Permeation enhancers, such as propylene glycol-lauric acid, have also proven to be successful in increasing transdermal delivery of lipophilic drugs and could, therefore, increase tazarotene’s permeation [35]. Additionally, chitosan presents an intriguing choice for enhancing drug permeability owing to its remarkable permeability-enhancing characteristics, favorable biocompatibility, and biodegradability [36]. The in vitro permeation study ultimately confirmed that tazarotene can permeate the skin in a detectable amount, and the addition of permeation enhancers could aid in increasing the amount of drug absorbed transdermally.

The SC is thought to be the primary rate-limiting step of transdermal drug absorption. Therefore, the tape-stripping method can be used to determine the penetration depth of drugs and indicate their concentration in deeper tissues [37]. The amount of tazarotene retained in the SC was identified through tape-stripping of the skin. As previously shown, the amount of the drug detected in the SC reduced with increased depth into the skin. This may be due to an increase in cohesion of cells with increased depth into the SC. Overall, 5% of the total amount of tazarotene applied to the skin was retained in the SC, further emphasizing the need for permeation enhancers. The tape-stripping analysis was effective in proving that a fraction of the substance remained in the SC. It is, however, likely that more of the drug remained in the deeper layers of the skin, especially considering tazarotene’s high lipophilicity [38]. Thus, it would be valuable to identify the amount of the drug remaining in the rest of the skin and subcutaneous fat, as well as collect data from more replicates.

The solution used for the transdermal drug release studies was challenging to apply to the porcine toe, therefore as an alternative, a cream including tazarotene was prepared for comparison to the gel. Tazarotene was present in the majority of examined joint samples. The concentration of analytes detected in the articular cartilage fluctuated between samples from the gel formulation, but less so in samples from the cream formulation. One possible limitation is the pH range of the two formulations. Creams typically have a higher lipid content than gels. This increased lipid concentration can be advantageous for lipophilic drugs, as it provides a better solubilizing environment and aids in their incorporation. Additionally, creams possess emollient properties, which can soften the stratum corneum, thus enhancing the penetration of lipophilic drugs. While the cream formulation yielded more consistent outcomes, additional investigation is required to refine the formulations. The cream was stable throughout this study, but further optimization and stability studies will be performed in future investigations. Various factors may have contributed to the variability in drug permeation observed with the gel formulation. As joints from different animals were used, variations in the permeation properties of the animal’s skin could have resulted in a higher standard deviation. Factors like skin thickness or structural differences can contribute to this variability [31]. Other factors that potentially had an impact on the results are the placement of the donor chambers, the amount of articular cartilage collected from the joints, and the degree of decomposition. Despite careful work, likely not all articular cartilage was collected from each joint. Thus, the sample only represents the articular cartilage that was removed and not the total amount of tazarotene delivered to the joints. The total amount of drug in the joint samples was less than the total permeated amount in the Franz diffusion cells. Nevertheless, this study offers important insights that can guide future research in the field. The findings confirm tazarotene’s ability to reach the joints in a detectable amount, providing useful resources for future research on tazarotene. The next steps will be additional analysis using a greater number of replicates, adjusting a suitable formulation, determining and mapping the drug delivery to the different skin layers with sectioning, and monitoring the travel from the surface of the stratum corneum to subcutaneous fat and articular cartilage in more detail. 

## 5. Conclusions

This study investigated the transdermal delivery potential of tazarotene, focusing on its application for joint-related conditions. Topical formulations, gel and cream bases, were investigated for their permeation through porcine skin and presence in articular cartilage. In vitro studies revealed detectible transdermal permeation of tazarotene in both solution and gel forms, with the gel showing a favorable stability profile, possibly due to ascorbic acid inclusion. Tape-stripping analysis indicated approximately 5% retention in the stratum corneum. The pilot study on porcine joints showed tazarotene reaching articular cartilage, but variability especially with the gel suggests further exploration. Challenges include skin thickness, structural differences, and donor chamber placement. Despite challenges, the findings confirm tazarotene’s potential to permeate the skin and reach joints, providing insights for future research. Optimization, the incorporation of permeation enhancers, and more extensive replication are crucial for a comprehensive understanding of tazarotene’s distribution within joint tissues and its potential therapeutic applications. 

## Figures and Tables

**Table 1:** List of ingredients for gel and cream formulations.

Gel		Cream	
Ingredient	Amount	Ingredient	Amount
Ascorbic acid	1.35%	Carbopol	1.08%
Carbopol	2.16%	Ceta-stearyl alcohol	10.83%
Distilled water	54.07%	Distilled water	75.78%
Natrium hydroxide 5 M	1.86%	Natrium hydroxide 5 M	1.49%
Polyethylene glycol 6′000	13.52%	Paraffin oil	10.83%
Propylene glycol	27.03%		

**Table 2:** Mass parameters for the analytes.

Compound	RT (min)	MW (*m/z*)	Product Ion (*m/z*)
Tazarotene	1.38	352.20	324.10
Tazarotenic acid	0.83	324.10	294.10
Linearity: 0.99885 Accuracy and precision: 97.25–100.98%, CV 3.20–7.43%			

**Table 3:** Tazarotene in articular cartilage of joints.

	Joint	Tazarotene (ng)	Tazarotenic Acid (ng)	Tazarotene Absorbed into Articular Cartilage (%) *
Gel	First joint	106.22 ± 95.90	38.48 ± 0 **	0.011%
	Second joint	122.06 ± 65.42	38.08 ± 1.47	0.012%
Cream	First joint	101.11 ± 0.12	45.59 ± 6.18	0.010%
	Second joint	270.09 ± 0.09	40.77 ± 1.96	0.027%

* The percentage of the topically applied tazarotene detected in the cartilage sample. ** Only one sample yielded results.

## Data Availability

The data presented in this study are available upon request from the corresponding author. The data are not publicly available at this time as they will be used in other ongoing studies.
